# Serum Cytokine Profile in Asian Indian Patients with Takayasu Arteritis and its Association with Disease Activity

**DOI:** 10.2174/1874312901711010023

**Published:** 2017-02-28

**Authors:** Ruchika Goel, Jayakanthan Kabeerdoss, Babu Ram, John Antony Jude Prakash, Sudhir Babji, Aswin Nair, Lakshmanan Jeyaseelan, Visalakshi Jeyaseelan, John Mathew, Veeraraghavan Balaji, George Joseph, Debashish Danda

**Affiliations:** 1 Department of Clinical Immunology and Rheumatology, Christian Medical College, Vellore-632004, Tamil Nadu, India; 2 Department of Biostatistics, Christian Medical College, Vellore-632004, Tamil Nadu, India; 3 Department of Cardiology, Christian Medical College, Vellore-632004, Tamil Nadu, India; 4 Wellcome Trust Research Laboratories, Division of Gastro Intestinal Sciences Christian Medical College, Vellore- 632004, Tamil Nadu, India; 5 Department of Clinical Microbiology, Christian Medical College, Vellore-632004, Tamil Nadu, India

**Keywords:** Takayasu arteritis, Large vessel vasculitis, Cytokines, Biomarkers, Interleukin-6, Interferon-gamma, Asian Indians

## Abstract

**Background:**

Arterial inflammation Takayasu arteritis (TA) is an outcome of balance between pro- and anti-inflammatory cytokines. Comprehensive assessment of these cytokines is important for understanding pathogenesis and assessing disease activity.

**Objective:**

To study pro- and anti-inflammatory cytokines representing different T-helper cell pathway in serum samples of Asian Indian patients with TA and to assess their association with disease activity.

**Methods:**

Consecutive Indian patients with TA were assayed for serum interferon-γ, interleukin-6, interleukin-23, interleukin-17, interleukin-10 and transforming growth factor- β levels at baseline and follow up visit. Patients were grouped into active and stable disease based on Indian Takyasu Arteritis clinical Activity Score-2010. Serum levels of these cytokines between active and stable disease and between baseline and follow up visits were compared by non-parametric tests.

**Results:**

Among 32 patients enrolled, 15 were classified as active while 17 as stable disease at baseline. IFN-γ levels were significantly higher in active disease than stable disease (p=0.0129) while other cytokines did not differ significantly between 2 groups. Serum levels of none of the cytokines changed significantly over 2 visits in both responders and non-responders. IL23 levels positively correlate with disease duration ((r=0.999; p<0.005). Modest correlation was observed between IFN-γ and IL23 levels at both baseline and follow up and between IFN-γ and IL-6 and CRP at follow up.

**Conclusion:**

IFN-γ levels are raised in active disease in TA and correlates well with other biomarkers of disease activity and proinflammatory cytokines. There is also a direct correlation between Il-23 levels and disease duration.

## INTRODUCTION

Takayasu arteritis (TA) is a rare, large-vessel vasculitis of unknown etiology affecting the aorta and main branches [1]. Cell-mediated immunity has been thought to predominate the initial inflammatory process which ultimately leads onto stenosis, occlusion and aneurysm formation in TA. Several pro-inflammatory cytokines such as tumor necrosis factor (TNF)-α, interferon (IFN)-γ, IL-6, IL-12 and IL-18 have been associated with granuloma formation on large vessels [2, 3]. Granuloma formation in giant cell arteritis (GCA), the other granulomatous large vessel vasculitis, has been clearly deciphered to involve the Th1 associated cytokines (IL-12, IFN-γ) as well as Th17 associated cytokines (IL-6, IL-17 and IL-23) [4]. Transforming growth factor (TGF)-β is a pro-fibrotic cytokine per se, but in the presence of pro-inflammatory cytokine IL-6, it induces *de novo* differentiation of IL-17 producing T cells [5].

The profile of T cells driving the inflammatory process and search for biomarkers is the focus of current research in TA, especially in the setting of a lack of ideal biomarkers to assess disease activity in this medical condition. Previous studies conducted in the Korean and Turkish populations have reported elevated levels of IL-6 and IL-18 among their TA patients [6, 7]. However, the cytokine profile has differed across studies from different regions of the world. This could be due to differences in clinical profile of disease across various series. For instance, TA patients in series from India had lesser female predominance (61%) than series from Japan and USA (91% to 96%). Pulmonary and renal arterial involvement is more common in Indian patients (49%, 53%) as compared to Japanese (4.7%, 21.7%) and Americans (7%, 18%). Fever as a symptom has been reported to be less common in Indians as compared to the other two ethnic cohorts [8-10].

Our aim was to study the relation between pro- and anti-inflammatory cytokines (IFN-γ, IL-6, IL-23, IL-17, IL-10 and TGF-β) representing different T-helper cell pathway in serum samples of Asian Indian patients with TA and to assess their association with disease activity.

## MATERIALS AND METHODS

Consecutive Indian patients satisfying the ACR criteria for TA were recruited from our outpatient and in-patient services [11]. TA patients were assessed according to disease activity by ITAS2010 (Indian Takayasu Arteritis Clinical Activity Score) and ITAS-A (CRP) [12]. ITAS 2010 ≥2 or ITAS-A (CRP) ≥3 was categorized as active disease while patients with ITAS 2010 =0 or ITAS-A CRP ≤2 were categorized as stable disease. ITAS 2010 of 1 and ITAS-A CRP of 2 was considered as indeterminate with respect to their disease activity status. Only those patients with unambiguous evidence of disease activity who could be classified into either active or inactive disease were included in the study. Patients with indeterminate disease activity were hence excluded from the study.

During follow up, patients were classified as responders and non-responders. Responders were those who had active disease at baseline but attained stable disease state during follow up. Inability to attain stable disease during follow up in a patient with active disease at baseline or a relapse into active disease in a patient with stable disease at baseline were grouped into non responders.

Blood samples of all recruited patients were collected at baseline and at follow-up. Serum cytokines IFN-γ, IL-23, TGF-β1 (Invitrogen Corporation, Camarillo, CA) and IL-10, IL-17 (BioSource Europe S.A., Nivelles Belgium.) levels were estimated using commercial Enzyme-Linked Immunosorbent Assay (ELISA) kit as per the manufacturer’s protocol.

### Statistical Analysis

Depending upon the type of data distribution, the data are depicted as mean ± S.D. or median (IQR). Mann Whitney U test was used for comparing the cytokine levels between two groups and Wilcoxon test were used to compare cytokine levels at 2 visits. Correlation between parameters was assessed by Spearman correlation test using GraphPad Prism software.

## RESULTS

 Thirty two consecutive Indian patients satisfying the ACR criteria for TA were recruited [11]. Among these, 15 patients were classified as active disease while 17 had stable disease at baseline visit.

Table **1** summarizes the demographic, angiographic and laboratory details of the subjects. Twenty nine (90.63%) patients were treated with steroids (mean dose: 27.24±18.53 prednisolone equivalent), 27 (87.1%) patients were on additional immunosuppressant (Mycophenolate in 23 patients and 4 were on Azathioprine).

At baseline, the active disease group had numerically higher levels of acute phase reactants (ESR and CRP) compared to stable disease group but it was not statistically significant. Median ITAS score of patients at baseline and follow-up were 0(0-6) and 0(0-0) respectively.

**Fig. (1):**
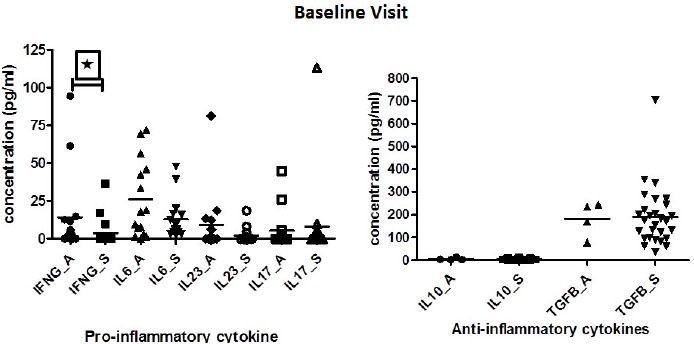
Represents the concentration of concentration of pro and anti-inflammatory cytokines in serum sample of TA patients (active (A) and stable (S)) at baseline (BL).

At baseline, however, IFN-γ levels were found to be significantly elevated in patients with active disease when compared to patients with stable disease (p=0.0129). IL-6 and IL-23 levels tended to be higher in patients with active disease as compared to stable TA patients, but did not attain statistically significance (p=0.3447). On the contrary, IL-10 and TGF-β levels were elevated among stable patients as compared to patients with active disease, but again didn’t attain statistical significance (Table **1**)(Fig. **1**).

IL23 levels were observed to be positively correlated with disease duration (r=0.999; p<0.005). IL17 levels were below measurable limits in most of the patients, so the results were not anlayzed further.

**Fig. (2):**
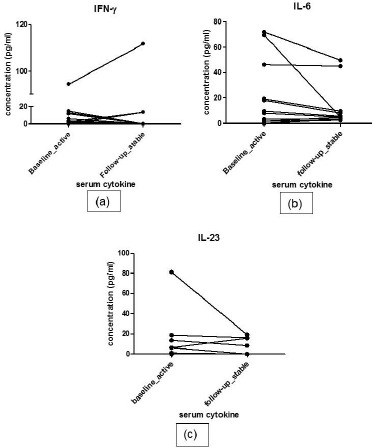
Represents the profile of pro-inflammatory cytokine profile in responders group. IFN-γ level at baseline and at follow-up (a). IL-6 level at baseline and at follow-up (b). IL-23 level at baseline and at follow-up (c).

At follow-up, 12 patients responded to treatment while 4 patients were classified as non-responders. The responders had decreased pro-inflammatory cytokine and increased anti-inflammatory cytokine profile. Serial estimation at 2 time points did not show significant difference in levels of any of the pro-inflammatory cytokines *i.e.* IFN-γ, IL-6 and IL-23 between 2 visits in responders (baseline *vs*. follow up: IFN-γ: 3.50(0.42-12.53) *vs*. 0(0-10.28)pg/ml, p=0.69; IL-6: 8(2-39) *vs*. 5.2(3.6-36.15) pg/ml, p= 0.285 ; IL-23:1(0-11.69) *vs*. 0(0-13.68)pg/ml, p=0.241). Nor did the levels of anti-inflammatory cytokines show any difference during follow up (Baseline *vs*. follow up- IL-10:4.1(2.8-6.2) *vs*. 1.4(0.-5.88) pg/ml, p=0.513; TGF-β: 153.8(76.33-232.2) *vs*. 125.1(86.01-189.4) pg/ml, p=0.969). IL-6 level and IL-23 increased numerically in non-responders at follow up visit (median values at baseline *vs*. follow up: IL-6 38.3(12.05-53.23) *vs*. 24.75(7.6-45.05) pg/ml; IL-23 2.1(0-10.43) *vs*. 2.1(0- 13.54), ; IFN-γ: 19.75(0.7-55.3) *vs*. 0(0-22.35)pg/ml,; IL-10 10.35(0.35-32.09) *vs*. 3.45(2.27-11.6) pg/ml,; TGF-β 159(36.93-286.4) *vs*. 203.3(101.7-242.6) (Fig. **2**). However, due to very small number of active patients (n=4) at follow-up in this group, statistical analysis was not performed.

We next determined the correlation between various cytokines studied and other inflammatory markers *i.e.*. ESR and CRP. At baseline, IFN- γ levels significantly correlated with IL-23 (r=0.389; p<0.05) and ESR (r=0.373;p<0.05). ESR had a good correlation with IL-6 and CRP also (r=0.506; p<0.005 and r=0.484; p<0.05 respectively). Similarly, at follow-up, a significant correlation was found between IFN-γ and CRP, IFN-γ and IL-6, IFN-γ and IL-23 (r= 0.427; p<0.05r=0.543; p<0.05, r=0.74; p<0.005 respectively).

## DISCUSSION

In this first ever Asian Indian study, we have evaluated all the three T-helper cell lineage cytokines in TA patients. The striking finding in this study were raised IFN-y levels in active patients at baseline and good correlation of this cytokine with conventional markers of disease activity (ESR an CRP) as well as with IL-6, the main pro-inflammatory cytokine shown previously to cause inflammation in TA. This observation becomes especially relevant in Indian TA patients in context of higher prevalence of tuberculosis in India. Various studies have postulated tuberculosis to be implicated in pathogenesis of TA and IFN-γ is the main cytokine responsible for granuloma formation both in TB and possibly in TA [13, 14].

Previously, Tripathy *et al.* have also shown an elevated mRNA expression of IFN-γ in TA patients [15]. Similarly, an increased mRNA expression level of IFN-γ has been observed in GCA [16, 17]. In addition, we observed numerically higher levels of IL-6 in serum of patients with active disease when compared to stable disease at baseline visit. Serum IL-6 level have been shown to be elevated in TA and GCA patients with active disease in a previous study by Salvarani *et al*. [18]. A very recent Japanese study has also observed an increase in the levels of IL-6 and TNF-α in TA patients during active phase [19]. However, in yet another study from Turkey, IL-18 but not IL-6 was found to be elevated in active disease [20]. Tocilizumab, an IL-6 R blocker has been shown to control inflammation in this disease [21]. Use of baseline immunosuppressant in majority of patients could have led to insignificant results in our study.

IL-10 regulates the immune response by suppressing the release and function of pro-inflammatory cytokines such as TNF-α, IL-1β and IL-6 [22]. A trend towards elevated levels of IL-10 and low levels of IL-6 in stable patients shows a that a critical balance of these cytokines may be an important factor in controlling inflammation in TA patients on treatment.

We also observed numerically higher levels of IL-23 in our patients with active disease. This is in line with other reports showing involvement of Th17 cytokines in TA in literature, as IL-23 is an activating cytokine of Th17 cells [23, 24]. Positive correlation between IL-23 and disease duration in our study possibly shows that Th17 cells may have proliferated over a longer course of disease. Mirsattari *et al*., too have reported a positive correlation between IL-23 and disease duration in ulcerative colitis, a reported co-morbidity in some cases of TA [25].

One of the main limitations of the present study is the small sample size of non-responders during follow-up due to which we could not clearly establish the importance of serially measuring these cytokines for disease activity assessment in TA. Studies with larger sample size are required for further understanding of role of these cytokines in pathogenesis and management of TA patients. Moreover, estimation of these cytokines in-vitro in supernatant of cultured PBMCs or tissue immunostaining would probably be better than serum estimation to assess the real cytokine profile of TA patients. The relation of IL-23 with disease duration also needs to be explored further.

## CONCLUSION

IFN-γ levels are raised in active disease in TA and correlates well with other biomarkers of disease activity and proinflammatory cytokines. Il-23 level also correlated with disease duration in TA.

## Figures and Tables

**Table 1:** Comparison of clinical, angiographic and laboratory features of TA patients at baseline and follow-up.

	**At Baseline**		**Follow up**	
	Active	Stable	Active	Stable
N	15	17	4	28
Median age in years (IQR)	24.0(20.0-29.0)	32.0(25.0-42.5)	24.0(20.3-45.8)	28.5(20.8-34.5)
Male/Female	1/14	2/15	0/4	3/25
Median disease duration prior to recruitment in months (IQR)	18.0(6.0-36.0)	24.0(12.0-54.0)	9.5(5.0-16.3)	6.5(3-10.75)
ITAS-2010	6.0(2.0-8.0)	0.0(0.0-0.0)	3.5(0.8-7.8)	0(0.0-0.0)
ESR (mm/1^st^ hr)	36.5(14.0-70.8)	20.0(13.5-43.0)	34(15-35)	20(12-39)
CRP (mg/L)	4.5 (1.1-33.2)	3.4(0.6-11.0)	15.6(6.11-18.0)	2.9 (0.73-12.3)
Angiographic assessment				
Type1:2:3:4:5 (n)	3: 0: 2: 2: 8	7: 0: 2: 3: 5	1: 0: 0: 0: 3	9: 0: 4: 5: 10
Serum Cytokines levels(pg/ml)				
IFN-γ**	2.8(0-12.8)	0(0-0)	0(0-22.35)	0(0-8.83)
IL-6*	18.2(3.2-46.2)	9.6(4.8-16.33)	24.85(7.6-45.05)	8.8(4.8-17.65)
IL-23*	1.0(0.0-12.5)	0.0(0.0-2.6)	2.1(0-13.54)	0(0-6.94)
IL-17*	0.0(0.0-2.0)	0.0(0.0-2.95)	3.2(0-10.38)	0(0-1.58)
IL-10*	1.4(0.0-6.9)	2.8(1.8-6.2)	3.45(2.28-11.6)	4.1(2.8-6.55)
TGF-β*	169.1(98.9-235.1)	206.3(78.9-249.1)	203.3(101.7-242.6)	173.1(96.6-237.9)

**p <0.01. *P values for IL-6, IL-23, IL-17, IL-10 and TGF-β are 0.3447, 0.1548, 0.7693, 0.2285 and 0.8799 respectively for comparison between active *vs.* stable disease group.
